# From social exclusion to interpersonal alienation among Chinese college students: the sequential mediating roles of social avoidance and self-disclosure inhibition

**DOI:** 10.3389/fpsyg.2026.1831369

**Published:** 2026-06-15

**Authors:** Yi Liu, Pan Chen, Peng Lei, Wei Tang

**Affiliations:** 1 School of Economics, Xihua University, Chengdu, China; 2 School of Tourism and Cultural Industries, Sichuan Tourism University, Chengdu, China; 3 School of Economics, Southwestern University of Finance and Economics, Chengdu, China

**Keywords:** Chinese college students, interpersonal alienation, self-disclosure inhibition, serial mediation, social avoidance, social exclusion

## Abstract

**Background:**

Social exclusion—being ignored, rejected, or excluded in social interactions—is a prevalent interpersonal stressor among college students, yet the specific mechanisms linking exclusion to long-term interpersonal difficulties remain poorly understood.

**Objective:**

This study tested a serial mediation model examining whether social avoidance and self-disclosure inhibition sequentially mediate the association between social exclusion and interpersonal alienation among Chinese college students.

**Methods:**

A total of 1,166 Chinese college students (64.67% female; *M* = 20.12, SD = 1.29) completed measures of social exclusion, social avoidance, self-disclosure, and interpersonal alienation. Serial mediation analysis using PROCESS Model 6 with bias-corrected bootstrap confidence intervals (5,000 resamples) was conducted.

**Results:**

The model explained 45.4% of the variance in interpersonal alienation. Social exclusion was significantly associated with interpersonal alienation (β = 0.571, *p* < 0.001). Three significant indirect pathways were identified: a behavioral pathway through social avoidance, a cognitive pathway through self-disclosure inhibition, and a serial pathway from social avoidance to self-disclosure inhibition. The behavioral and cognitive pathways contributed approximately equally, while the serial pathway contributed a smaller but significant portion. Results remained robust after controlling for gender and academic level.

**Conclusions:**

The findings support a sequential pattern in which social exclusion is associated with behavioral avoidance, which is linked to reduced self-disclosure and greater interpersonal alienation. This pattern suggests that interventions targeting both behavioral avoidance and self-disclosure inhibition may help address exclusion-related interpersonal difficulties among college students.

## 1 Introduction

Social exclusion—the experience of being ignored, rejected, or excluded by individuals or groups in social interactions (Williams and Nida, 2022)—is a prevalent interpersonal stressor with significant implications for psychological wellbeing and social functioning (Riva et al., 2017). While extensive research has documented the negative consequences of exclusion experiences, the current understanding of the specific mechanisms through which exclusion is associated with long-term interpersonal difficulties remains incomplete (Baumeister and Leary, 1995; Smart Richman and Leary, 2009). This gap in mechanistic understanding limits both theoretical advancement and practical intervention development. The present study addresses this limitation by proposing and testing a serial mediation model that examines how social exclusion is associated with interpersonal alienation—characterized by emotional disconnection from others, perceived social isolation, and difficulty forming meaningful relationships (Yang and Wu, 1998)—through sequential behavioral and cognitive response patterns among Chinese college students. Specifically, we investigate whether social exclusion is associated with behavioral avoidance of social interaction, which may in turn be linked to reduced self-disclosure, ultimately predicting greater interpersonal alienation.

### 1.1 Social exclusion: conceptualization and consequences

Social exclusion encompasses both overt forms, such as explicit rejection and deliberate ostracism, and more subtle forms, such as being overlooked or left out of group activities. Both forms threaten fundamental psychological needs including belonging, self-esteem, control, and meaningful existence (Williams and Nida, 2022). Baumeister and Leary's (1995) seminal belongingness theory established that the need to belong constitutes a basic human motivation, and that threats to belonging are associated with powerful psychological and behavioral responses aimed at restoring social connection. While some degree of social exclusion is inherent to group dynamics and in-group/out-group differentiation, persistent or pervasive exclusion from valued social groups has been consistently associated with significant adverse outcomes (Smart Richman and Leary, 2009).

Research has documented a trajectory of consequences following social exclusion, from acute emotional distress to longer-term interpersonal difficulties. In the immediate aftermath of exclusion, individuals typically experience physiological pain responses, need threat, and emotional distress (Williams and Nida, 2022). When individuals are unable to restore their sense of belonging, these acute responses may develop into more persistent patterns, including alienation, depression, helplessness, and feelings of being unworthy of attention (Williams and Nida, 2022). This trajectory is particularly concerning among college students, who face heightened social comparison processes and intensive peer interaction demands—including dormitory relationships, class group dynamics, and extracurricular social networks—during the critical developmental period of emerging adulthood (Arnett, 2000). Recent research among Chinese college students has highlighted the severity of these concerns, with exclusion experiences being associated with psychological crisis vulnerability (Li and Li, 2026) and elevated social anxiety, with gender differences observed in both the experience and consequences of exclusion (Shuai et al., 2024).

Despite this growing body of research documenting the consequences of social exclusion, significant gaps remain in the current understanding of the specific mechanisms through which exclusion experiences are associated with long-term interpersonal difficulties. The majority of existing research has focused on identifying outcomes rather than elucidating processes, limiting the ability to develop targeted interventions and theoretical models that capture the complexity of exclusion responses (Williams and Nida, 2011, 2022). Understanding the specific pathways through which exclusion is associated with interpersonal alienation can inform the development of targeted interventions that address underlying processes rather than merely symptoms.

### 1.2 Social avoidance: behavioral responses to exclusion

Social avoidance behavior represents one of the most immediate and observable responses to social exclusion, serving as a behavioral response pattern that minimizes exposure to further interpersonal threats. From an adaptive perspective, avoidance behaviors can provide immediate protection from additional rejection experiences while allowing individuals time to recover emotionally (Maner et al., 2007). However, avoidance becomes maladaptive when it generalizes beyond the specific rejecting context to novel social situations in which acceptance would otherwise be possible, thereby reducing opportunities for new connections and ultimately exacerbating the very interpersonal difficulties it was initially designed to address.

This overgeneralization process operates through multiple interconnected mechanisms that transform initially adaptive responses into maladaptive patterns of interpersonal dysfunction. Social learning theory provides a useful framework for understanding how avoidance behaviors become entrenched through reinforcement mechanisms. When individuals avoid social situations following exclusion experiences, they typically experience immediate relief from social anxiety, creating negative reinforcement that strengthens avoidance tendencies (Bandura and Walters, 1977). Simultaneously, the absence of positive social experiences prevents the development of approach behaviors and social confidence.

Empirical research has provided substantial support for the association between social exclusion and subsequent avoidance behaviors across diverse populations and methodological approaches. Laboratory studies using experimental exclusion paradigms consistently demonstrate increased avoidance intentions and behaviors following exclusion manipulations (Molden et al., 2009), with cross-cultural evidence indicating that these effects are robust across cultural contexts (Williams et al., 2000). Recent ecological research has further confirmed the real-world relevance of these findings: experience sampling studies show that avoidance and withdrawal are predominant behavioral responses to everyday ostracism (Büttner et al., 2025), and converging experimental evidence establishes that ostracism consistently increases preferences for solitude (Ren et al., 2021). In the Chinese cultural context specifically, social exclusion has been shown to predict social withdrawal behavior among adolescents (Chen et al., 2023). Longitudinal research has also been valuable in establishing that exclusion experiences predict increases in avoidance behaviors over time (London et al., 2007).

The Chinese cultural context presents unique considerations for understanding these associations, as collectivistic values and social harmony emphases may influence both the expression and consequences of avoidance behaviors. Chinese cultural norms often promote conflict avoidance and face-saving behaviors that may make social withdrawal more socially acceptable than in individualistic cultures (Chen et al., 2006). Recent cross-cultural research has highlighted the importance of cultural factors in shaping responses to social exclusion, with Chinese populations showing distinct patterns of behavioral and emotional regulation compared to Western samples (He et al., 2021).

### 1.3 Self-disclosure inhibition: cognitive responses to social threat

Self-disclosure represents a fundamental mechanism through which individuals develop and maintain intimate relationships, serving as both a tool for intimacy regulation and a vehicle for social connection (Reis and Shaver, 1988). Research consistently demonstrates that self-disclosure quantity and quality predict relationship satisfaction, stability, and depth across various relationship types (Collins and Miller, 1994). Among college students specifically, self-disclosure quality has been shown to be associated with perceived social support and reduced loneliness through serial mediation pathways, underscoring its importance as a key interpersonal mechanism (Guo et al., 2026). Under conditions of social threat, however, individuals appear to adopt more cautious disclosure strategies that prioritize self-protection over relationship development. Self-disclosure inherently involves vulnerability, as sharing personal information creates opportunities for judgment, rejection, and exploitation by others (Omarzu, 2000). When individuals perceive elevated social threat following exclusion experiences, they may strategically reduce disclosure tendencies to minimize exposure to additional interpersonal risks.

Emerging empirical evidence directly supports the association between social exclusion and reduced self-disclosure. Ji et al. (2026) demonstrated through a series of experimental studies that individuals exposed to exclusion reported significantly lower self-disclosure than those in inclusion conditions, with interpersonal trust mediating this effect. These findings suggest that exclusion may erode the trust necessary for open communication, thereby constraining disclosure willingness. Complementing this experimental evidence, Meral et al. (2021) found that individuals who experienced rejection anticipated social costs rather than benefits when considering whether to disclose that experience, feeling reluctant to share despite an urge to do so. This pattern indicates that disclosure inhibition following exclusion is not simply an absence of motivation to communicate, but rather a strategic response to perceived interpersonal risks.

The consequences of disclosure inhibition for relationship quality and interpersonal functioning are significant. Reduced self-disclosure directly impairs relationship development by preventing the gradual intimacy increases that characterize healthy relationship progression (Laurenceau et al., 1998). When individuals withhold personal information and emotional expression, relationship partners may interpret this behavior as disinterest or emotional unavailability, potentially contributing to reciprocal withdrawal that further compromises relationship quality. Research on social exclusion further indicates that exclusion experiences are associated with withdrawal-oriented regulatory responses, including reduced self-disclosure and restrained social sharing (Smart Richman and Leary, 2009). Individual differences in cognitive control capacity and emotion regulation strategies may moderate the association between exclusion and disclosure inhibition (Downey and Feldman, 1996).

The cultural context of Chinese society presents additional considerations for understanding disclosure inhibition patterns, as traditional Chinese values emphasize emotional restraint, interpersonal harmony, and careful consideration of self-presentation in social contexts (Bond and Hwang, 1986; Ip et al., 2021). In the Chinese online context, Zeng and Zhu (2021) found that fear of evaluation—a construct closely related to social threat perception—was negatively associated with the amount of self-disclosure among Chinese social media users, and that this association was strengthened by protective face orientation, a culturally salient concern about losing face. These findings suggest that in Chinese collectivistic contexts, the tendency to inhibit self-disclosure following perceived social threat may be amplified by cultural norms regarding face and social evaluation (Fitzpatrick et al., 2006).

### 1.4 Serial mediation model: theoretical rationale

The present study proposes a serial mediation model that conceptualizes social avoidance and self-disclosure inhibition as sequential mediating mechanisms linking social exclusion to interpersonal alienation. This theoretical framework represents a departure from existing single-mechanism models that typically examine either behavioral or cognitive responses to exclusion in isolation. The serial mediation approach posits that behavioral responses to social threats may subsequently be associated with changes in cognitive processes, forming a sequential pattern that may progressively undermine interpersonal functioning (Smith and DeCoster, 2000). Specifically, while belongingness theory established the fundamental importance of social connection needs, it provided limited insight into how initial responses to exclusion may develop into entrenched patterns of interpersonal dysfunction (Baumeister and Leary, 1995). The serial mediation model addresses this gap by proposing that behavioral avoidance may serve as an antecedent to self-disclosure inhibition: as individuals repeatedly withdraw from social contexts following exclusion, they may not only reduce immediate exposure to rejection but also progressively limit opportunities for self-disclosure, with this skill atrophy and reinforced beliefs about the risks of vulnerability potentially transforming temporary withdrawal into persistent barriers against intimacy.

The rationale for proposing a sequential rather than merely independent mediation rests on three converging lines of evidence. First, recent time-course research provides direct support for the temporal priority of behavioral responses following exclusion. Kip et al. (2025) using time-contingent sampling to track coping responses at Day 0, Day 3, and Day 6 after naturally occurring ostracism experiences, found that individuals initially prioritized withdrawal as a coping response, with more varied cognitive coping strategies emerging only at later time points. This temporal pattern is consistent with experience sampling research demonstrating that behavioral avoidance and withdrawal are the predominant responses when psychological needs are severely threatened by ostracism (Büttner et al., 2025). Together, these findings suggest that under conditions of significant social threat, behavioral withdrawal represents a temporally prior response that precedes more complex cognitive adjustments (Sherman, 2013; Vaillant, 1992).

Second, empirical research supports the proposed mechanistic link from avoidance to disclosure inhibition. Sun and Jakubiak (2024) demonstrated that avoidant interpersonal orientations are associated with dramatically reduced personal event sharing in close relationships—a one scale-point increase in avoidance predicted a 68% decrease in the odds of sharing—and that sharing in more avoidant contexts became selectively restricted to low-vulnerability content. Although this research focused on attachment avoidance rather than exclusion-induced avoidance specifically, the pattern is conceptually consistent: avoidant tendencies constrain both the quantity and vulnerability of interpersonal disclosure. This constraint may operate through both an opportunity reduction mechanism, wherein withdrawal limits the contexts in which self-disclosure could occur, and a risk recalibration process, wherein withdrawal reinforces perceptions that disclosure is risky (Meral et al., 2021).

Third, recent research among Chinese college students provides converging support for serial mediation models linking social exclusion to downstream outcomes through sequential psychological mechanisms. Zhang et al. (2025) demonstrated that social exclusion was associated with social anxiety through the chain mediation of fear of negative evaluation and rejection sensitivity, establishing that exclusion can activate sequential psychological processes in this population. The present study extends this line of research by proposing that behavioral and cognitive mechanisms may also operate sequentially, with social avoidance preceding and constraining self-disclosure in the pathway from exclusion to interpersonal alienation.

Importantly, the serial mediation model does not preclude the possibility that social avoidance and self-disclosure inhibition also operate as independent mediators. Both mechanisms may be directly associated with exclusion while simultaneously maintaining a sequential relationship. This integrated perspective recognizes that exclusion may be associated with both immediate behavioral withdrawal and cognitive self-protection, while the behavioral response subsequently amplifies the cognitive adjustment. It should also be noted that the proposed directional sequence—from exclusion to avoidance to disclosure inhibition—represents a theoretically grounded hypothesis rather than a confirmed causal chain. Alternative directional models, including the possibility that pre-existing social withdrawal or disclosure reluctance may contribute to individuals being excluded, remain plausible and warrant investigation through longitudinal and experimental designs.

**Figure 1:**
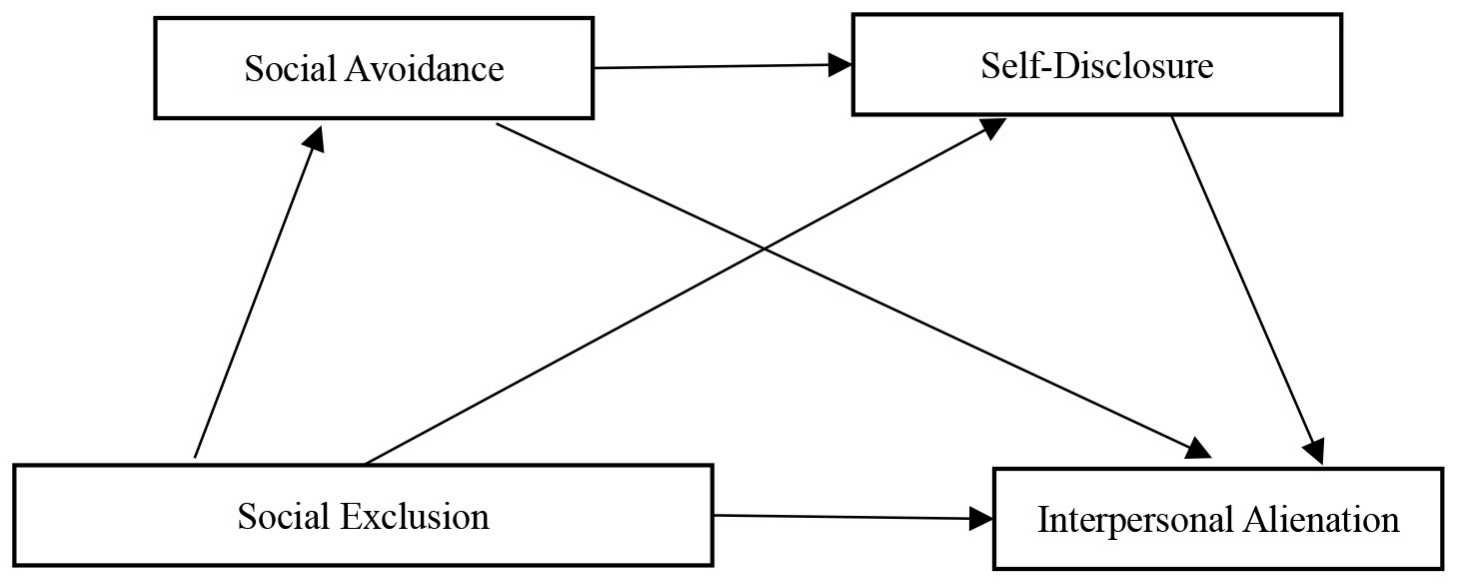
Theoretical model framework.

As shown in Figure 1, the theoretical model depicts the hypothesized serial mediation relationship between social exclusion and interpersonal alienation. The model illustrates two direct mediation pathways through social avoidance (behavioral mechanism) and self-disclosure inhibition (cognitive mechanism), with an additional sequential pathway linking social avoidance to self-disclosure inhibition, representing the hypothesized sequential pattern wherein behavioral withdrawal is subsequently associated with constrained self-disclosure opportunities.

Based on the theoretical foundation and empirical evidence reviewed above, the present study tests four specific hypotheses:

Hypothesis 1 (H1): Social exclusion will be significantly and positively associated with interpersonal alienation.

Hypothesis 2 (H2): Social avoidance will significantly mediate the association between social exclusion and interpersonal alienation. Specifically, higher levels of social exclusion will positively predict increased social avoidance, which will in turn positively predict greater interpersonal alienation.

Hypothesis 3 (H3): Self-disclosure inhibition will significantly mediate the association between social exclusion and interpersonal alienation. Specifically, higher levels of social exclusion will predict decreased self-disclosure, which will in turn be associated with greater interpersonal alienation.

Hypothesis 4 (H4): Social avoidance and self-disclosure inhibition will sequentially mediate the association between social exclusion and interpersonal alienation. Specifically, higher levels of social exclusion will be associated with increased social avoidance, which will in turn be associated with reduced self-disclosure, ultimately predicting greater interpersonal alienation.

## 2 Method

### 2.1 Participants and procedure

#### 2.1.1 Sampling strategy and target population

The present investigation employed a stratified sampling design targeting undergraduate students across universities in Sichuan Province, China. Specifically, five universities were purposefully selected to represent different institutional types within the province, including two applied vocational colleges, two general comprehensive universities, and one “Double First-Class” (National Key) university. This composition broadly reflects the structural distribution of higher education institutions in China, enhancing the diversity of the sample. Within each institution, stratified sampling ensured proportional representation across academic levels and major academic fields. College students represent a theoretically relevant population for examining social exclusion and interpersonal alienation mechanisms due to the heightened social comparison processes, identity formation challenges, and intensive peer interaction contexts characteristic of emerging adulthood (Arnett, 2000). The focus on this population does not imply that college students are uniquely or optimally suited for examining these processes, but rather that the social dynamics of this developmental period—including frequent transitions in peer networks and dormitory-based communal living—make exclusion experiences and their interpersonal consequences particularly salient (Li and Li, 2026).

#### 2.1.2 Data collection procedures

Data were collected between July and September 2025 via an online survey platform. Survey links were distributed through university communication channels within the five selected universities. Before beginning the questionnaire, each participant was required to read a detailed informed consent form and provide explicit agreement, confirming their understanding of the study's purposes, procedures, and confidentiality protections. Participants were also informed of their right to withdraw from the study and request deletion of their data at any time. The estimated completion time was 15–20 min. Participation was entirely voluntary, with no academic or financial incentives provided to minimize coercion concerns.

#### 2.1.3 Sample characteristics and quality control

The final analytic sample comprised 1,166 Chinese college students ranging in age from 18 to 25 years (*M* = 20.12, SD = 1.29). The sample included 754 female participants (64.67%) and 412 male participants (35.33%). Academic level distribution was: freshman (*n* = 476, 40.8%), sophomore (*n* = 293, 25.1%), junior (*n* = 377, 32.3%), and senior (*n* = 20, 1.7%). The low proportion of seniors likely reflects reduced on-campus availability due to off-campus internship and employment activities during the data collection period. Monthly living expenses were distributed as follows: ≤ 1000 RMB (*n* = 176, 15.1%), 1001–2000 RMB (*n* = 860, 73.8%), 2001–3000 RMB (*n* = 101, 8.7%), and >3000 RMB (*n* = 28, 2.5%).

Data quality control procedures included multiple validation checks consistent with recommended practices for survey-based research (Meade and Craig, 2012; Curran, 2016). Participants completing surveys in less than 10 min or more than 30 min were excluded (*n* = 92), as response times outside this range suggest either insufficient engagement or significant interruptions during completion. Attention check items embedded throughout the survey identified inattentive responding (*n* = 48 excluded). Response pattern analyzes excluded participants showing excessive straight-lining or impossible response combinations (*n* = 34 excluded). The initial recruitment yielded 1,340 participants, resulting in an 87.0% retention rate following quality control procedures. Exclusion decisions were based primarily on response quality indicators (completion time and response consistency) rather than content-based criteria, thereby balancing data integrity with the avoidance of excessive participant filtering. Comparisons between excluded and retained participants on demographic variables (gender, age, academic level) revealed no significant differences (all ps > 0.05), indicating that the quality control procedures did not systematically bias the composition of the analytic sample.

#### 2.1.4 Ethical considerations and approval

All procedures received approval from the Sichuan Psychology Association Ethics Review Committee, ensuring compliance with Chinese psychological research ethics guidelines and international standards. The informed consent process, as described in Data collection procedures Section, ensured that all participants were fully informed of their rights prior to participation. No deception was employed, and all measures assessed naturalistic social experiences.

#### 2.1.5 Statistical power and sample size adequacy

Power analysis for serial mediation models using Monte Carlo simulation methods (Schoemann et al., 2017) indicated that detecting small to medium indirect effects required approximately 550 participants for 80% power at α = 0.05. The achieved sample size (*N* = 1,166) provided >99% power for detecting the hypothesized mediation effects and >85% power for comparing relative pathway contributions. Bootstrap confidence interval procedures require substantial sample sizes for stable estimation, and the current sample size ensures reliable performance for complex mediation models (Hayes and Scharkow, 2013).

### 2.2 Measures

All measures were administered in Chinese. Each scale and its scoring approach used in the present analyzes are described below. The specific constructs assessed were social exclusion (predictor), social avoidance (first mediator), self-disclosure (second mediator, reverse-scored to index inhibition), and interpersonal alienation (outcome). All scales used in this study are well-established instruments with demonstrated reliability and validity in Chinese populations.

#### 2.2.1 Social exclusion scale

Social exclusion was assessed using a Chinese version of the social exclusion questionnaire specifically developed for Chinese college students by Wu et al. (2013). The scale was originally adapted from Western exclusion measures and culturally modified to capture both direct and indirect forms of social exclusion relevant in Chinese collectivistic contexts (Chen et al., 2006). The 19-item scale comprises two dimensions: direct exclusion (10 items; e.g., “Other students have deliberately ignored me in social situations,” “I have been explicitly excluded from group activities”) and indirect exclusion (9 items; e.g., “I often feel left out of group activities even when I'm physically present,” “Others seem to overlook my presence in social settings”). The inclusion of indirect exclusion items is consistent with established ostracism research, which recognizes that subjective experiences of being overlooked or left out—even in the absence of explicit rejection—constitute a meaningful and frequently studied form of social exclusion (Williams and Nida, 2022). This dual-focus approach is particularly important in Chinese interpersonal contexts where indirect exclusion may be more culturally normative than direct rejection.

Participants responded using a 5-point Likert scale ranging from 1 (never) to 5 (always), with higher total scores indicating greater exclusion experiences. The total score across all 19 items was used in the present analyzes. The scale demonstrated excellent internal consistency in the current sample (α = 0.89). The original validation study reported strong test-retest reliability over a 2-week interval (*r* = 0.84; Wu et al., 2013). Confirmatory factor analysis supported a two-factor structure distinguishing direct and indirect exclusion (CFI = 0.94, TLI = 0.93, RMSEA = 0.067, SRMR = 0.048). Convergent validity was established through significant correlations with loneliness (*r* = 0.67) and social anxiety measures (*r* = 0.58).

#### 2.2.2 Social avoidance subscale

Social avoidance behavior was measured using the Social Avoidance subscale from the Social Avoidance and Distress Scale (SADS) (Watson and Friend, 1969), validated for Chinese student populations by Peng et al. (2003). The Chinese version demonstrated robust psychometric properties and cultural appropriateness for assessing avoidance behaviors in Chinese college contexts. The 14-item subscale specifically assesses behavioral tendencies to avoid social interaction and withdraw from interpersonal situations (e.g., “I try to avoid situations which require me to be very sociable,” “I usually go out of my way to avoid trouble or disagreements,” “I often make excuses to avoid social gatherings”). This measure focuses exclusively on overt avoidance behaviors rather than accompanying emotional distress, providing a pure assessment of the behavioral component independent from anxiety or discomfort.

Response options ranged from 0 (strongly disagree) to 4 (strongly agree), with higher total scores reflecting greater avoidance tendencies. The total score across all 14 items was used in the present analyzes. The subscale exhibited strong internal consistency in the current sample (α = 0.86) and demonstrated excellent construct validity through factor analyzes confirming independence from the distress component of the original scale. Discriminant validity was supported by moderate correlations with social anxiety (*r* = 0.45) while maintaining distinctiveness from social fears and evaluative concerns, confirming that the subscale measures behavioral avoidance rather than affective responses.

#### 2.2.3 Self-disclosure scale

Self-disclosure was assessed using the Chinese adaptation of the Self-Disclosure Index (Miller et al., 1983), validated by Chen (2020) for adult Chinese populations. The adapted version maintains the theoretical focus on disclosure breadth and depth while incorporating culturally appropriate contexts for Chinese interpersonal relationships. The 15-item scale evaluates both the extent and intimacy level of personal information sharing across various relationship contexts (e.g., “I usually tell others about my personal problems and difficulties,” “I often discuss my feelings and emotions with close friends,” “I openly share my thoughts about family matters with others”). Items assess disclosure across emotional, experiential, and opinion domains, providing comprehensive coverage of self-disclosure propensity.

Participants rated their typical disclosure patterns using a 5-point scale from 1 (not at all characteristic) to 5 (very characteristic). The total score approach was selected to capture the overall propensity for openness and vulnerability in relationships, representing the theoretical construct of interest rather than specific disclosure domains. The total score across all 15 items was used in the present analyzes; lower scores indicate greater self-disclosure inhibition as conceptualized in the mediation model. The total score demonstrated high internal consistency in the current sample (α = 0.83) and strong convergent validity with relationship intimacy measures (*r* = 0.71). Factor analyzes supported a general disclosure factor underlying specific content domains, justifying the total score approach. This scoring strategy enhances statistical power for mediation analyzes while maintaining theoretical coherence (Greene et al., 2006).

#### 2.2.4 Interpersonal alienation scale

Interpersonal alienation was measured using the Interpersonal Alienation subscale from the Alienation Scale developed by Yang and colleagues for Chinese student populations (Yang and Wu, 1998; Zhang and Yang, 2003). Although this instrument was originally developed and validated with adolescent samples, it has been subsequently used in research with Chinese college students (Zeng et al., 2022; Hu et al., 2022), and the item content addresses interpersonal experiences (emotional disconnection, perceived isolation, difficulty forming relationships) that are equally relevant across late adolescence and emerging adulthood. This instrument was specifically designed to assess alienation experiences within Chinese cultural contexts, addressing unique interpersonal dynamics and social expectations characteristic of collectivistic cultures. The 14-item subscale assesses three interrelated dimensions of interpersonal alienation: emotional disconnection from others (e.g., “I feel emotionally distant from people,” “I have difficulty feeling warmth toward others”), perceived social isolation (e.g., “I often feel like an outsider in social groups,” “I feel separated from those around me”), and difficulties forming meaningful relationships (e.g., “It's hard for me to feel really close to people,” “I struggle to develop deep connections with others”). This multidimensional approach captures the complex nature of interpersonal alienation while distinguishing it from related constructs such as loneliness or social anxiety.

Items were rated on a 5-point Likert scale from 1 (strongly disagree) to 5 (strongly agree), with higher total scores indicating greater interpersonal alienation. The total score across all 14 items was used in the present analyzes. The scale showed excellent reliability in the current sample (α = 0.91) and strong construct validity through confirmatory factor analysis supporting the three-factor structure (CFI = 0.96, TLI = 0.95, RMSEA = 0.058), confirming the appropriateness of this instrument for the present college student sample. Convergent validity was demonstrated through substantial correlations with loneliness (*r* = 0.78) and negative correlations with social support measures (*r* = −0.65), while discriminant validity was supported by moderate correlations with depression (*r* = 0.52), indicating distinctiveness from general psychological distress.

### 2.3 Data analysis strategy

Data analysis began with comprehensive preliminary analyzes including descriptive statistics for all study variables and correlation analyzes to examine bivariate relationships among social exclusion, social avoidance, self-disclosure, and interpersonal alienation. Common method bias was assessed using Harman's single-factor test, which revealed that the first unrotated factor accounted for 32.7% of the total variance, well below the 40% threshold indicating absence of serious common method bias. Multicollinearity was evaluated using variance inflation factor (VIF) calculations, with all values below 5.0 indicating acceptable levels of intercorrelation among predictors.

Serial mediation analysis using PROCESS Model 6 (Hayes, 2018) was selected as the primary analytical approach based on theoretical considerations proposing a sequential relationship between behavioral and cognitive response patterns. It should be noted that PROCESS employs a regression-based approach and does not yield traditional structural equation modeling fit indices (e.g., CFI, RMSEA); therefore, the model's explanatory utility is evaluated through variance explained (*R*^2^) for each endogenous variable rather than overall model fit statistics. The serial mediation framework allows simultaneous examination of multiple mediating pathways while testing their relative contributions, directly addressing our central research question regarding the relative roles of behavioral and cognitive mechanisms. Indirect effects were estimated using bias-corrected bootstrap procedures with 5,000 resamples to generate robust confidence intervals. Bootstrap methods provide superior performance for mediation analysis by avoiding distributional assumptions and providing accurate Type I error rates for indirect effects (MacKinnon et al., 2004). Significance testing relied exclusively on bias-corrected 95% confidence intervals, with effects considered significant when intervals exclude zero. To examine the robustness of the mediation results after accounting for demographic characteristics, we also ran the serial mediation model with gender and academic level included as covariates. Additionally, supplementary multi-group analyzes were conducted to explore potential gender differences in the mediation pathways.

## 3 Results

### 3.1 Preliminary analysis

Prior to conducting the primary analyzes, comprehensive data screening procedures were implemented to ensure the integrity and appropriateness of the dataset for serial mediation analysis. Missing data analysis revealed no systematic patterns of missingness across the core study variables, with complete data available for all 1,166 participants.

Assumption testing for multiple regression and mediation analysis was conducted following established guidelines (Hayes, 2018; Preacher and Hayes, 2008). Examination of residual plots indicated that assumptions of linearity, homoscedasticity, and independence were satisfactorily met. Multicollinearity diagnostics revealed variance inflation factors (VIF) ranging from 1.120 to 1.890, well below the conventional threshold of 5.000, indicating acceptable levels of predictor intercorrelation (Hair et al., 2010). Normality assessments using the Kolmogorov–Smirnov test and visual inspection of Q–Q plots confirmed that all variables approximated normal distributions, with skewness and kurtosis values falling within acceptable ranges (|skewness| < 2.000, |kurtosis| < 7.000; West et al., 1995).

Descriptive statistics and intercorrelations among study variables are presented in Table 1. Social exclusion scores ranged from 19 to 95 (*M* = 35.920, SD = 13.806), indicating substantial variability in exclusion experiences across the sample. Social avoidance scores were generally low (*M* = 3.691, SD = 2.367), while self-disclosure total scores (*M* = 61.424, SD = 7.582) and interpersonal alienation scores (*M* = 39.321, SD = 9.770) demonstrated moderate levels with adequate dispersion for analysis.

**Table 1:** Descriptive statistics and intercorrelations among study variables.

Variable	*M*	SD	1	2	3
1. SE	35.920	13.806	–		
2. SA	3.691	2.367	0.193^***^	–	
3. SD	61.424	7.582	−0.194^***^	−0.212^***^	–
4. IA	39.321	9.770	0.574^***^	0.366^***^	−0.398^***^

*N* = 1,166.

SE, Social Exclusion; SA; Social Avoidance; SD, Self-Disclosure; IA, Interpersonal Alienation.

^***^p < 0.001.

The correlation matrix revealed patterns consistent with the hypothesized model. Social exclusion exhibited significant positive correlations with social avoidance (*r* = 0.193, *p* < 0.001) and interpersonal alienation (*r* = 0.574, *p* < 0.001), while demonstrating a significant negative correlation with self-disclosure (*r* = −0.194, *p* < 0.001). Social avoidance showed significant positive associations with interpersonal alienation (*r* = 0.366, *p* < 0.001) and negative correlations with self-disclosure (*r* = −0.212, *p* < 0.001). Self-disclosure exhibited the expected negative correlation with interpersonal alienation (*r* = −0.398, *p* < 0.001). These bivariate associations were consistent with the proposed mediation pathways and warranted proceeding with the serial mediation analysis. It should be noted, however, that the correlational patterns are also consistent with alternative directional models and do not themselves confirm the hypothesized sequence.

### 3.2 Serial mediation model testing

The serial mediation model was tested using PROCESS Model 6 (Hayes, 2018) with bootstrap confidence intervals based on 5,000 resamples. The model accounted for 45.4% of the variance in interpersonal alienation (*R*^2^ = 0.454), representing a substantial proportion of explained variance (Cohen, 2013). Complete path coefficients and model summaries for each regression equation are presented in Table 2.

**Table 2:** Path coefficients and model summary for each regression equation.

Outcome variable	Predictor	*B*	SE	*t*	*p*	95% CI	β	*R^2^*	*F*
SA								0.034	38.650^***^
	Constant	2.556	0.195	13.140	< 0.001	[2.174, 2.938]	—		
	SE	0.032	0.005	6.220	< 0.001	[0.022, 0.042]	0.184		
SD								0.063	37.100^***^
	Constant	66.556	0.658	101.120	< 0.001	[65.265, 67.848]	—		
	SE	−0.084	0.016	−5.150	< 0.001	[−0.116, −0.052]	−0.153		
	SA	−0.554	0.095	−5.840	< 0.001	[−0.740, −0.368]	−0.174		
IA								0.454	304.420^***^
	Constant	44.836	2.072	21.640	< 0.001	[40.771, 48.902]	–		
	SE	0.339	0.016	21.040	< 0.001	[0.308, 0.371]	0.483		
	SA	0.864	0.094	9.150	< 0.001	[0.679, 1.049]	0.211		
	SD	−0.338	0.030	−11.430	< 0.001	[−0.396, −0.280]	−0.263		

SE, Social Exclusion, SA, Social Avoidance; SD, Self-Disclosure; IA, Interpersonal Alienation.

CI, confidence interval.

^***^p < 0.001.

As shown in Table 2, all hypothesized pathways were statistically significant. Social exclusion significantly predicted increased social avoidance (β = 0.184) and decreased self-disclosure (β = −0.153). Social avoidance was significantly associated with reduced self-disclosure (β = −0.174), consistent with the proposed sequential link between the two mediators. Both mediators were significantly associated with the outcome: social avoidance positively predicted interpersonal alienation (β = 0.211), while self-disclosure negatively predicted interpersonal alienation (β = −0.263). Detailed statistics for all paths, including unstandardized coefficients, standard errors, and confidence intervals, are reported in Table 2.

### 3.3 Hypothesis testing

The total effect of social exclusion on interpersonal alienation was significant (*B* = 0.401, SE = 0.017, 95% CI [0.367, 0.435], β = 0.571), providing support for Hypothesis 1. This finding indicates that social exclusion is robustly associated with increased interpersonal alienation among Chinese college students. Following the inclusion of mediators, the direct effect remained significant but was reduced (β = 0.483), indicating partial mediation. The total indirect effect was significant (*B* = 0.062, SE = 0.010, 95% CI [0.044, 0.082], β = 0.088), indicating that the mediators collectively account for a meaningful portion of the association between social exclusion and interpersonal alienation. Complete mediation results are presented in Table 3.

**Table 3:** Serial mediation analysis results: social exclusion to interpersonal alienation.

Effect	*B*	SE	95% CI	β	% of total
Total effect	0.401	0.017	[0.367, 0.435]	0.571	–
Direct effect	0.339	0.016	[0.308, 0.371]	0.483	–
Total indirect	0.062	0.010	[0.044, 0.082]	0.088	100.00%
Individual pathways					
Behavioral: SE → SA → IA	0.027	0.006	[0.017, 0.039]	0.039	44.40%
Cognitive: SE → SD → IA	0.028	0.007	[0.016, 0.043]	0.040	46.00%
Serial: SE → SA → SD → IA	0.006	0.002	[0.003, 0.010]	0.008	9.60%

SE, Social Exclusion; SA, Social Avoidance; SD, Self-Disclosure; IA, Interpersonal Alienation. Bootstrap confidence intervals based on 5,000 samples.

As shown in Table 3, all three individual indirect pathways were statistically significant. The behavioral mediation pathway (social exclusion → social avoidance → interpersonal alienation) yielded a significant indirect effect (*B* = 0.027, 95% CI [0.017, 0.039]), supporting Hypothesis 2. This finding indicates that higher levels of social exclusion are associated with greater avoidance behaviors, which in turn are associated with greater interpersonal alienation. The cognitive mediation pathway (social exclusion → self-disclosure inhibition → interpersonal alienation) also yielded a significant indirect effect (*B* = 0.028, 95% CI [0.016, 0.043]), supporting Hypothesis 3. This finding indicates that social exclusion is associated with reduced self-disclosure tendencies, which in turn are associated with increased interpersonal alienation. The sequential mediation pathway (social exclusion → social avoidance → self-disclosure inhibition → interpersonal alienation) revealed a significant indirect effect (*B* = 0.006, 95% CI [0.003, 0.010]). Although smaller in magnitude, the confidence interval excludes zero, supporting Hypothesis 4. This finding is consistent with the proposed sequential pattern in which social exclusion is associated with behavioral avoidance, which in turn is associated with reduced self-disclosure, ultimately predicting greater interpersonal alienation.

Bootstrap confidence intervals for all indirect effects were calculated using bias-corrected methods (MacKinnon et al., 2004), with all 95% confidence intervals excluding zero. The behavioral pathway contributed 44.4% of the total indirect effect, the cognitive pathway contributed 46.0%, and the sequential pathway contributed 9.6%. Statistical comparison of the behavioral and cognitive pathways revealed no significant difference (*B* = −0.001, 95% CI [−0.015, 0.013]), indicating approximately equivalent contributions from both primary mediation pathways. This balanced pattern suggests that social exclusion is associated with both behavioral and cognitive response patterns, rather than being preferentially associated with one mechanism over the other.

**Figure 2:**
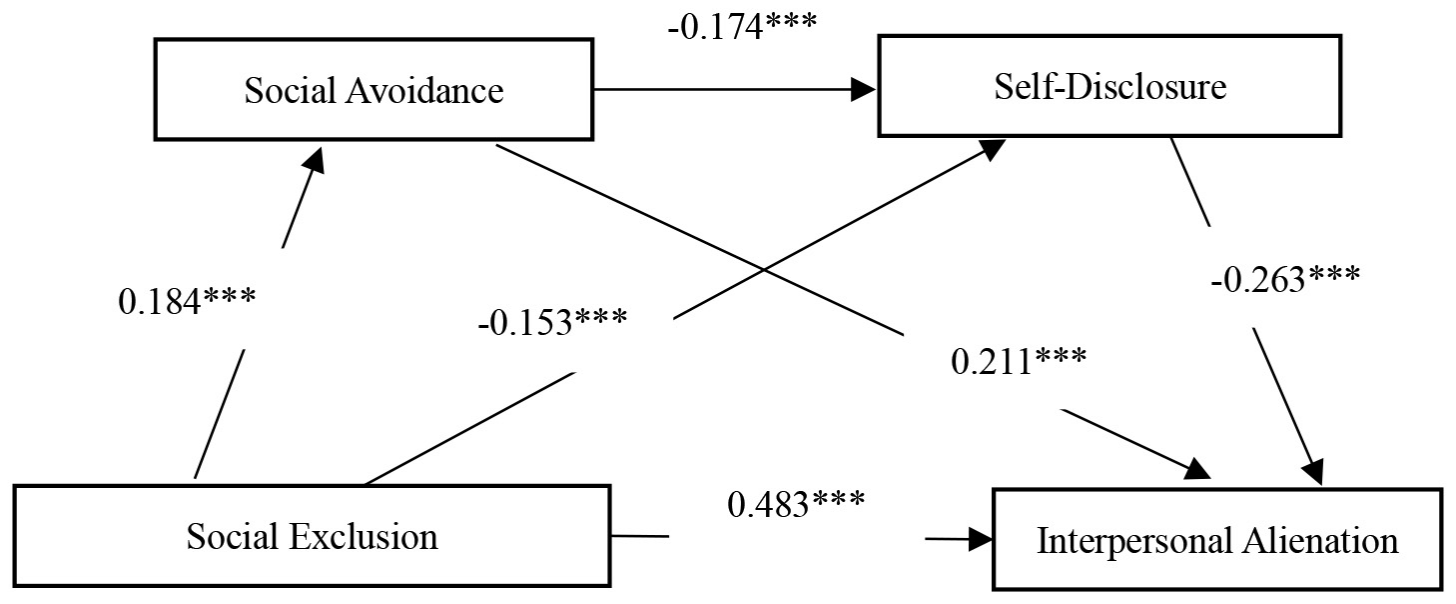
Serial mediation model with standardized path coefficients. ^***^p < 0.001.

As shown in Figure 2, serial mediation model examining the associations among social exclusion, social avoidance, self-disclosure inhibition, and interpersonal alienation (*N* = 1,166). Path coefficients are standardized beta weights (β = 0.184 for SE → SA, β = −0.153 for SE → SD, β = −0.174 for SA → SD, β =0.483 for SE → IA direct effect, β = 0.211 for SA → IA, β = −0.263 for SD → IA). Solid lines represent significant paths (*p* < 0.001). The model explained 45.4% of variance in interpersonal alienation. Total effect: β = 0.571. All four hypotheses were supported.

### 3.4 Supplementary analyzes

To examine the robustness of the mediation results after accounting for demographic characteristics, the serial mediation model was re-run with gender and academic level included as covariates. Neither gender nor academic level significantly predicted any of the endogenous variables in the model (all ps > 0.05). The pattern of results remained essentially unchanged: the total effect (*B* = 0.407), direct effect (*B* = 0.341), and total indirect effect (*B* = 0.066) were highly consistent with the primary model, confirming that the mediation findings are robust after accounting for these demographic variables.

Additionally, the serial mediation model was estimated separately for female (*n* = 754) and male (*n* = 412) participants to explore potential gender differences. In both subsamples, the total effect of social exclusion on interpersonal alienation was significant (female: *B* = 0.420; male: *B* = 0.372; both ps < 0.001), and the direct effect remained significant after including mediators (female: *B* = 0.359; male: *B* = 0.284; both ps < 0.001). The total indirect effect was significant in both groups (female: *B* = 0.061, 95% CI [0.041, 0.085]; male: *B* = 0.087, 95% CI [0.046, 0.141]), and all three individual indirect pathways reached significance with bootstrap confidence intervals excluding zero. The model explained substantial and comparable proportions of variance in interpersonal alienation across genders (female: *R*^2^ = 0.447; male: *R*^2^ = 0.484). These results suggest that the overall pattern of mediation is consistent across male and female participants, supporting the generalizability of the findings within the present sample.

## 4 Discussion

### 4.1 Principal findings and theoretical contributions

The present investigation provides findings consistent with a serial mediation model linking social exclusion to interpersonal alienation among Chinese college students. The most significant theoretical contribution lies in showing that behavioral avoidance and self-disclosure inhibition may operate as both independent and sequential mediating mechanisms. Specifically, social avoidance and self-disclosure inhibition each contributed approximately equal proportions to the total indirect effect through direct pathways, while the serial pathway from social avoidance to self-disclosure inhibition contributed an additional portion, suggesting a sequential pattern wherein behavioral responses may subsequently be associated with changes in cognitive processes. The model explained a substantial proportion of variance in interpersonal alienation (*R*^2^ = 0.454), and the large association between exclusion experiences and interpersonal alienation, combined with the significant but reduced direct effect following the inclusion of mediators, suggests that behavioral and cognitive mechanisms account for meaningful portions of this association.

These findings extend Baumeister and Leary's (1995) belongingness theory by identifying specific mechanisms through which thwarted belonging needs may manifest as interpersonal dysfunction. Recent reviews have noted that prolonged ostracism is associated with resignation, alienation, and feelings of unworthiness (Williams and Nida, 2022), yet the specific pathways linking exclusion to these outcomes have received limited empirical attention. The present study addresses this theoretical gap by demonstrating that exclusion is associated with sequential behavioral and cognitive response patterns that are, in turn, associated with compromised interpersonal connections. The serial mediation finding moves beyond single-mechanism theoretical frameworks, suggesting that social threats are associated with behavioral responses that may subsequently constrain cognitive processes, forming a sequential pattern that may progressively undermine interpersonal functioning (Smith and DeCoster, 2000). This pattern is consistent with recent research among Chinese college students demonstrating that social exclusion can be associated with downstream outcomes through sequential psychological mechanisms, such as the chain mediation from exclusion through fear of negative evaluation and rejection sensitivity to social anxiety (Zhang et al., 2025).

Supplementary analyzes supported the robustness and generalizability of these findings within the present sample. The mediation results remained essentially unchanged when gender and academic level were included as covariates, and the overall pattern of mediation was consistent across male and female subsamples, with all indirect pathways reaching significance in both groups. These findings suggest that the associations identified in the primary model are not attributable to demographic confounds and that the sequential mediation pattern operates similarly for male and female Chinese college students.

### 4.2 Behavioral pathway: social avoidance as a response to exclusion

The significant behavioral mediation pathway supports the proposition that social avoidance represents a key behavioral response associated with social exclusion and a significant predictor of interpersonal alienation. This finding aligns with recent research demonstrating that social avoidance serves as a common response under conditions of interpersonal threat, particularly when psychological needs are severely threatened (Büttner et al., 2025). Converging evidence has further established that ostracism consistently increases preferences for solitude and withdrawal across multiple studies (Ren et al., 2021), and that social exclusion is associated with increased social withdrawal behavior in Chinese cultural contexts specifically (Chen et al., 2023).

The results are noteworthy in the Chinese cultural context, where social harmony and face-saving behaviors are paramount (Hu et al., 2024). The association between exclusion and behavioral withdrawal may be particularly salient in collectivistic settings where behavioral withdrawal can function as a culturally normative response that minimizes further interpersonal conflict. However, the association between avoidance and interpersonal alienation illustrates the potentially maladaptive consequences of these initially adaptive responses. When avoidance generalizes from the specific rejecting context to broader social situations, prolonged avoidance patterns may ultimately reduce opportunities for positive social interaction and skill development (Hofmann, 2007). Research among Chinese college students has further demonstrated that social avoidance is associated with increased loneliness and reduced self-control (He and Wang, 2025), underscoring its broader maladaptive correlates beyond the specific outcome examined here.

The substantial contribution of the behavioral pathway to the total indirect effect suggests that exclusion-related avoidance represents a primary mechanism that could be targeted through intervention approaches focusing on graduated exposure and behavioral activation strategies (Hofmann and Otto, 2008; Sherman, 2013). Moreover, as the proposed antecedent in the serial pathway, behavioral avoidance may play an initiating role in the sequential process associated with interpersonal alienation.

### 4.3 Cognitive pathway: self-disclosure inhibition as a response to social threat

The cognitive mediation pathway indicates that self-disclosure inhibition is an equally important mechanism associated with the link between social exclusion and interpersonal alienation. This finding extends intimacy regulation theory by suggesting how perceived social threats may alter processes of relationship development and maintenance. Recent experimental evidence directly supports this association: Ji et al. (2026) demonstrated that individuals exposed to exclusion conditions reported significantly lower self-disclosure than those in inclusion conditions, with interpersonal trust mediating this effect. This pattern is consistent with the theoretical proposition that exclusion may erode the sense of trust and safety necessary for open communication, thereby constraining disclosure willingness.

The theoretical significance of self-disclosure inhibition lies in its potential disruption of natural relationship progression patterns. Reis and Shaver (1988) intimacy process model emphasizes that relationship development depends on reciprocal self-disclosure cycles. Exclusion experiences may interrupt this progression by promoting self-protective strategies that prioritize risk avoidance over relationship advancement, potentially trapping individuals between conflicting needs for connection and protection. Meral et al. (2021) provided a compelling illustration of this conflict, demonstrating that individuals who experienced rejection felt an urge to disclose yet simultaneously felt reluctant to do so, because they anticipated social costs rather than benefits from sharing their experience. The importance of self-disclosure in interpersonal functioning is further underscored by research showing that disclosure quality is associated with perceived social support and reduced loneliness among college students (Guo et al., 2026).

Cultural considerations provide additional insight into the cognitive pathway. In the Chinese context, where evaluative threat and face concerns are particularly salient, fear of evaluation has been found to be negatively associated with the amount of self-disclosure among Chinese social media users, with this association strengthened by protective face orientation (Zeng and Zhu, 2021). Traditional Chinese values emphasizing emotional restraint may thus amplify the tendency toward disclosure inhibition following exclusion experiences (Chen et al., 2013).

### 4.4 Serial mediation mechanism: sequential behavioral-cognitive pattern

Further analysis suggests that social avoidance may serve as an antecedent of self-disclosure inhibition, with these two mechanisms potentially constituting a sequential pathway through which social exclusion is progressively associated with undermined interpersonal functioning. The serial pathway contributed a smaller but statistically significant portion of the total indirect effect, suggesting several theoretically important dynamics.

First, the finding is consistent with recent time-course evidence indicating that behavioral responses temporally precede cognitive adjustments following ostracism. (Kip et al. 2025) demonstrated through time-contingent sampling that individuals initially prioritized withdrawal as a coping response after naturally occurring ostracism, with more varied cognitive coping strategies emerging only at later time points. This temporal pattern supports the proposed sequence in which behavioral avoidance may precede and set the stage for subsequent cognitive adjustments, including reduced self-disclosure. Second, the proposed opportunity reduction mechanism may operate through a behavioral constraint process. Social avoidance may directly reduce the contexts in which self-disclosure could occur. (Sun and Jakubiak 2024) provided relevant evidence that avoidant interpersonal orientations are associated with dramatically reduced sharing of personal events in close relationships, and that sharing in more avoidant contexts becomes selectively restricted to low-vulnerability content. Although their focus was on attachment avoidance rather than exclusion-induced avoidance specifically, the pattern is conceptually consistent: avoidant tendencies may constrain both the quantity and vulnerability of interpersonal disclosure. When individuals systematically avoid social interactions, they may inherently limit opportunities to practice self-disclosure, potentially contributing to skill atrophy and reinforced beliefs about the risks of vulnerability. Third, a reinforcement process may perpetuate the sequential pattern. As behavioral avoidance reduces social contact, individuals may experience fewer opportunities for positive interpersonal experiences that might counteract exclusion-related concerns. The absence of corrective social experiences may allow negative expectations about the risks of self-disclosure to persist and intensify. This process is consistent with the finding that anticipated social costs are a key factor in disclosure reluctance following rejection (Meral et al., 2021).

It should be emphasized that these dynamics are proposed theoretical interpretations of the cross-sectional associations observed in the present study. The sequential pattern identified through mediation analysis is consistent with the hypothesized direction but does not confirm causal ordering. The serial mediation pattern is particularly relevant in the Chinese cultural context, where behavioral conformity and emotional restraint are culturally valued. Chinese college students experiencing exclusion may find behavioral withdrawal socially acceptable, potentially setting in motion the sequential process that is ultimately associated with interpersonal alienation. This cultural dimension underscores the importance of culturally sensitive interventions that recognize how normative behavioral patterns may be associated with maladaptive sequential processes.

### 4.5 Practical implications

The serial mediation findings suggest the potential value of sequenced intervention approaches addressing both behavioral and cognitive components of exclusion responses. Interventions focusing exclusively on either behavioral modification or cognitive restructuring may achieve suboptimal outcomes by failing to address the potentially sequential nature of these mechanisms. The results suggest that interventions could prioritize early behavioral engagement to address avoidance patterns before cognitive adjustments become entrenched, while also incorporating cognitive strategies to address disclosure inhibition that may already be established.

At the behavioral level, graduated exposure and behavioral activation strategies could be employed to disrupt avoidance patterns while building positive social experiences (Hofmann and Otto, 2008). The substantial contribution of the behavioral pathway and its proposed role as the antecedent in the serial pathway suggest that early behavioral interventions could potentially address both the direct association with alienation and the downstream cognitive associations. Research has suggested that promoting social engagement may enhance psychological wellbeing among college students who exhibit social avoidance tendencies (He and Wang, 2025). At the cognitive level, trust-building exercises and vulnerability training could address self-disclosure inhibition. However, the serial pathway findings suggest that cognitive interventions may be most effective when combined with behavioral activation, as avoidance may limit opportunities to apply newly developed disclosure skills. Research on self-disclosure in Chinese contexts further suggests that interventions should attend to cultural norms around face and evaluative concerns that may amplify disclosure reluctance (Zeng and Zhu, 2021).

Beyond individual-level intervention, prevention programs could incorporate early identification strategies targeting behavioral warning signs such as social withdrawal and reduced participation in group activities. Given that social exclusion among Chinese college students has been associated with a range of adverse outcomes including social anxiety (Shuai et al., 2024) and psychological crisis vulnerability (Li and Li, 2026), early identification and intervention may have broad mental health benefits. Educational institutions could also implement universal prevention programs that teach adaptive coping strategies as alternatives to behavioral withdrawal (Hofmann, 2007) and create environments that normalize self-disclosure and provide safe opportunities for social connection—for example, through inclusive dormitory environments and structured peer interaction within academic settings, which represent the primary social contexts in which Chinese college students experience exclusion.

### 4.6 Limitations and future directions

Several methodological limitations warrant consideration when interpreting these findings. Most critically, the cross-sectional design precludes definitive causal or temporal inferences about the relationships among social exclusion, mediating mechanisms, and interpersonal alienation. While the theoretical rationale supports the proposed sequence and the serial mediation analysis provides findings consistent with the sequential pathway, these results do not confirm causal ordering. Alternative directional models remain plausible—for example, individuals who are dispositionally inclined toward social withdrawal or disclosure reluctance may be more likely to experience exclusion as a consequence of these tendencies rather than as a cause. Longitudinal and experimental research is essential to test whether behavioral avoidance temporally precedes self-disclosure inhibition and whether both are consequences rather than antecedents of exclusion experiences. The time-course approach employed by Kip et al. (2025), who tracked coping responses at multiple time points following naturally occurring ostracism, offers a particularly promising methodological model for future studies.

Self-report measurement methodology introduces potential common method bias that may influence observed relationships among study variables. Although Harman's single-factor test suggested that common method bias was not a serious concern in the present data, shared method variance may contribute to the pathway effects observed. Future research should incorporate multi-method assessment approaches, including behavioral observations, peer reports, and experience sampling methods to provide convergent validation. The sample was drawn exclusively from universities in Sichuan Province, Southwestern China, which limits generalizability to other Chinese regions with different economic, cultural, and educational characteristics. The low representation of senior students (1.7%) further limits the generalizability of findings across all academic stages. Cultural factors may significantly influence exclusion responses, potentially limiting applicability to Western or other cultural populations. The developmental period of emerging adulthood may not generalize to younger adolescents or older adults with established social networks. Although supplementary analyzes indicated that the mediation pattern was consistent across male and female participants, the gender ratio in the present sample (64.67% female) may not fully represent the broader college student population, and future research with more balanced samples is warranted.

Future research should prioritize several specific directions. First, prospective longitudinal studies could examine whether behavioral avoidance at Time 1 predicts self-disclosure inhibition at Time 2, which in turn predicts interpersonal alienation at Time 3, thereby testing the proposed sequential pattern with temporal separation. Second, experimental studies systematically manipulating exclusion experiences while measuring behavioral and cognitive responses at multiple time points could provide stronger evidence regarding directionality. Third, intervention studies testing whether early behavioral activation is associated with reduced downstream cognitive inhibition could both test the sequential hypothesis and inform clinical practice (Hughes et al., 2017). Fourth, cross-regional and cross-cultural comparison studies would help determine which aspects of the sequential pathway are universal and which are culture-specific. Fifth, future research could examine the role of specific moderating factors—such as rejection sensitivity (Downey and Feldman, 1996), resilience (Shuai et al., 2024), and face orientation (Zeng and Zhu, 2021)—in determining which individuals are most vulnerable to the proposed sequential pattern.

## 5 Conclusion

The present study tested a serial mediation model linking social exclusion to interpersonal alienation among 1,166 Chinese college students, finding that behavioral avoidance and self-disclosure inhibition function as both independent and sequential mediating mechanisms. The serial pathway suggests a pattern wherein behavioral withdrawal may be associated with constrained self-disclosure opportunities, potentially undermining interpersonal connectedness—extending single-mechanism theoretical approaches by offering a framework for understanding how exclusion-related interpersonal difficulties may unfold through sequential behavioral and cognitive processes. These findings suggest the potential value of sequenced intervention approaches that address behavioral avoidance alongside self-disclosure inhibition, rather than targeting either process in isolation. Important limitations include the cross-sectional design, which precludes causal inferences, and the geographic restriction to Sichuan Province, which limits generalizability. The proposed sequential pattern represents a theoretically grounded hypothesis that requires confirmation through longitudinal and experimental research across diverse cultural contexts and developmental populations.

## Data Availability

The raw data supporting the conclusions of this article will be made available by the authors, without undue reservation.
